# Ventricular Arrhythmia Associated With Magnesium and Vitamin D Deficiencies in a Patient With Rheumatoid Arthritis

**DOI:** 10.7759/cureus.19143

**Published:** 2021-10-30

**Authors:** Han Joon Bae

**Affiliations:** 1 Internal Medicine, Cardiology, Daegu Catholic University Hospital, Daegu, KOR

**Keywords:** steroid use, serum magnesium, vitamin-d deficiency, qtc prolongation, ventricular dysrhythmia

## Abstract

Prolongation of QT associated with electrolyte changes can lead to ventricular arrhythmias. Correction and supply of calcium, magnesium, and potassium are essential to managing this condition. In this report, we present a case of QT prolongation due to magnesium and vitamin D deficiency in a patient with rheumatoid arthritis.

## Introduction

Glucocorticoids are routinely used to treat various diseases. One of the main indications for glucocorticoid therapy is inflammatory rheumatic disorders. However, prolonged glucocorticoid use is associated with hypocalcemia as well as magnesium and vitamin D deficiencies [1,2], and vitamin D and magnesium deficiencies also cause hypocalcemia. Hypocalcemia and magnesium deficiency can result in QT interval prolongation, predisposing the patient to life-threatening ventricular arrhythmias [3,4].

## Case presentation

**Figure 1:**
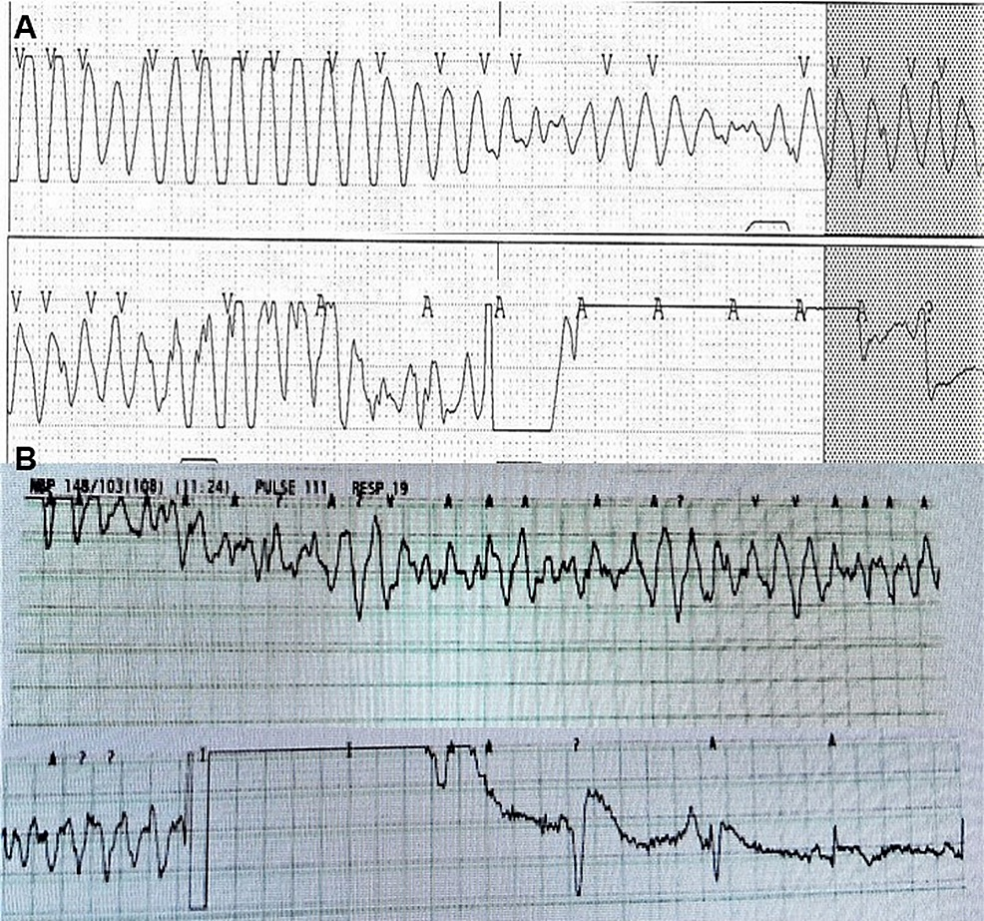
Wide QRS tachycardia A. Wide QRS tachycardia, torsade de pointes; B. Recurrent wide QRS tachycardia

A 60-year-old man presented to the emergency department of our institution with palpitations and an altered mental state due to ventricular tachycardia. His medical history included pneumoconiosis, nontuberculous mycobacteria lung disease, chronic obstructive lung disease, heart failure, and rheumatoid arthritis. He had no family history of cardiovascular disease, syncope, or sudden cardiac death. The patient was afebrile, with a pulse rate of 180 beats per minute, blood pressure of 83/50 mmHg, oxygen saturation of 95% with 6 L per minute of nasal cannula, respiratory rate of 25 breaths per minute, and there was no peripheral edema. Auscultation of the chest revealed diffuse bilateral crackles. Cardiac examination revealed an irregular rhythm without murmurs. The electrocardiogram on presentation demonstrated torsade de pointes (Figure 1).

**Figure 2:**
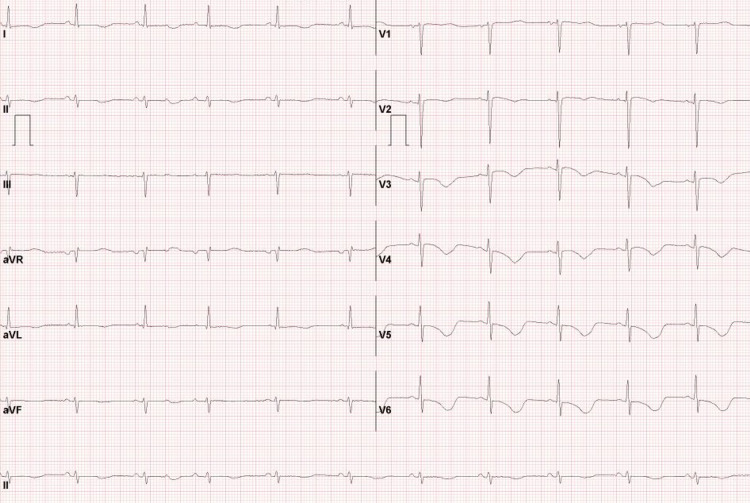
Electrocardiogram after defibrillation Prolonged QTc (571 ms)

**Figure 3:**
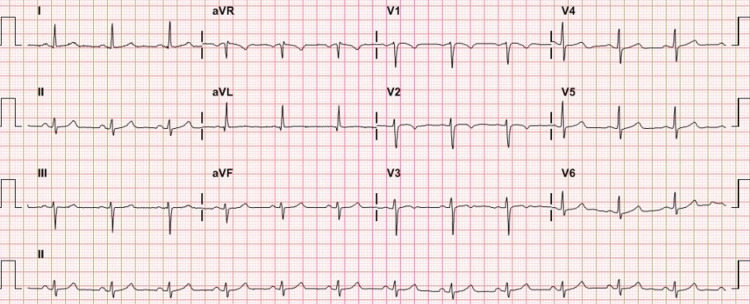
Previous electrocardiogram showing normal QTc

After defibrillation, an electrocardiogram indicated T inversion on V2-6, left anterior hemiblock, and prolonged QTc (571 ms) (Figure 2). During the hospitalization, despite intravenous infusions of calcium gluconate and magnesium sulfate, prolonged QTc and magnesium deficiency still persisted. The previous electrocardiogram had shown normal QTc (Figure 3). The echocardiography findings before and during the hospitalization showed no significant dysfunction (Table 1).

**Table 1:** Echocardiography LVD: left ventricular diameter; IVST: interventricular septum thickness; LAD: left atrial diameter; EF: ejection fraction; E: early diastolic velocity of mitral inflow; e': early diastolic velocity of mitral annular motion; RVSP: right ventricular systolic pressure

During admission				Previous (one month before admission)			
	Diastolic	Systolic	Unit		Diastolic	Systolic	Unit
LVD	46.9	32.2	mm	LVD	45	33	mm
IVST	8.9	12.2	mm	IVST			mm
LAD		28.3	mm	LAD		29	mm
EF	4-chamber	63.9	%	EF	4-chamber	46.0	%
	2-chamber	59.3	%		2-chamber		%
E/e'		7.5		E/e'			
RVSP		34	mmHg	RVSP			mmHg

The patient's medications included meloxicam 7.5 mg, methylprednisolone 4 mg, sulfasalazine 500 mg bid, montelukast 10 mg, valsartan 80 mg, bisoprolol 1.25 mg, pitavastatin 2 mg, and inhaled fluticasone (Avamys® nasal spray). Laboratory test results showed a pro B-type natriuretic peptide level of 3,315 pg/mL (reference range: <150 pg/mL), creatine kinase-MB level of 7.6 ng/mL, troponin-T level of 0.2 ng/mL, C-reactive protein level of 25.5 mg/dL (reference range: <5.0 mg/dL), phosphorous level of 4.6 mg/dL (reference range: 2.5-4.5 mg/dL), potassium level of 3.4 mmol/L (reference range: 3.5-5.1 mmol/L), calcium level of 7.6 mg/dL (reference range: 8.2-10.2 mg/dL), ionized calcium level of 1.19 mg/dL (reference range: 1.13-1.32 mg/dL), and magnesium level of 1.1 mg/dL (reference range: 1.5-2.7 mg/dL). His thyroid function test results and other laboratory data were within the respective normal ranges.

**Figure 4:**
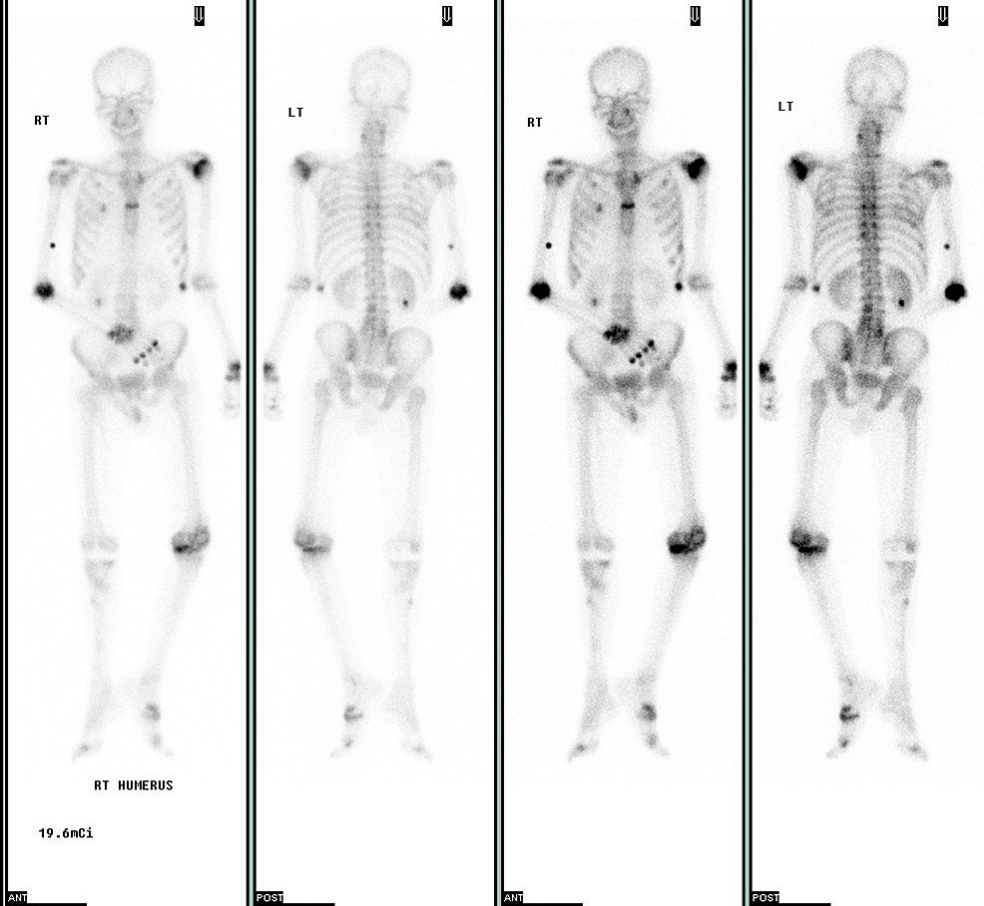
Bone scan Black lesion shows increased uptake due to arthritis

Based on these findings, we considered it important to evaluate his vitamin D, parathyroid hormone, and calcitonin levels. However, urine magnesium levels could not be tested in our hospital. Laboratory test results showed calcitonin level of 18.29 pg/mL (reference range: 0.9-9.5 pg/mL), parathyroid hormone level of 13 pg/mL (reference range: 10-65 pg/mL), 25-OH vitamin D level of 14 ng/mL (reference range - deficiency: <10, inadequacy: 10-30 ng/mL), and 1 α,25(OH)2 vitamin D level of 3.24 pg/mL (reference range: 19.6-54.3 pg/mL). The bone scan showed increased uptake due to arthritis (Figure 4). And bone mineral density showed osteopenia (Table 2).

**Table 2:** Bone mineral density (BMD) T-sore at or above -1.0 SD = normal; T-score between -1.0 and -2.5 SD = osteopenia; T-score at or below -2.5 SD = osteoporosis

Region	BMD (g/cm^2^)	Young adult		Age-matched	
		%	T-score	%	Z-score
L1	0.791	80	-1.6	87	-0.7
L2	0.829	83	-1.4	90	-0.5
L3	0.919	88	-1.1	95	-0.3
L4	0.940	90	-0.8	98	-0.1
L1-2	0.809	84	-1.2	92	-0.5
L1-3	0.859	87	-1.0	95	-0.3
L1-4	0.871	86	-1.0	94	-0.4
L2-3	0.878	86	-1.2	93	-0.4
L2-4	0.890	52	-1.3	92	-0.5
L3-4	0.93	86	-1.2	94	-0.4
Femur					
Neck	0.827	98	-0.2	114	0.8
Ward	0.814	114	0.8	160	2.4
Troch	0.714	101	0.1	106	0.4
Shaft	1.066	98	-0.1	104	0.2
Total	0.936	100	0.0	107	0.4

**Figure 5:**
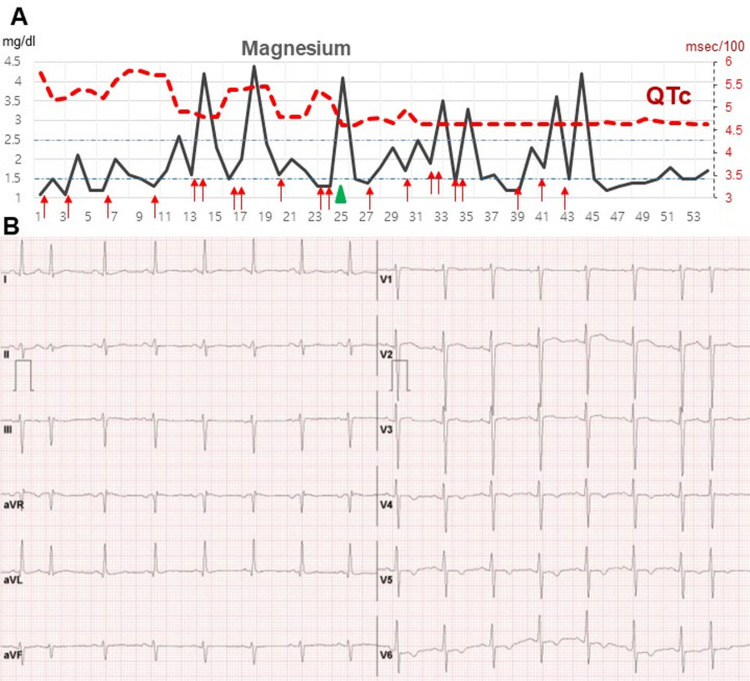
Changes in magnesium level and QTc A. Changes in magnesium level and QTc. B. Electrocardiography after discharge (one month later). The red arrow indicates intravenous magnesium supplementation, and the green arrow indicates vitamin D supplementation. Serum magnesium reference range: 1.5-2.7 mg/dL

After vitamin D supplementation (cholecalciferol 1,000 IU for 1.5 months), magnesium level and QTc were within normal limits. Changes in serum magnesium level and QTc are shown in Figure 5. And the electrocardiography after discharge (one month later) showed that QTc was within normal range.

## Discussion

The rheumatoid arthritis inflammatory reaction adversely affects both local joint and systemic bone remodeling [5]. Continuous use of corticosteroid therapy is associated with rapid bone loss and an increased risk of fractures [6,7]. Glucocorticoids, which reduce the absorption of calcium, appear to activate the transport of magnesium. Vitamin D may activate magnesium absorption, but its role remains unclear.

In the present case, the patient had many comorbidities, including rheumatoid arthritis, and despite the continuous supply of magnesium, calcium, and potassium, QTc prolongation and magnesium deficiency were not well corrected. Several causes of magnesium deficiency have been investigated, and the possibility of vitamin D playing a role may be considered. After vitamin D supplementation, a relatively stable magnesium level was maintained, and QTc was corrected.

Magnesium is mostly absorbed in the distal end of the small intestine, through the saturable transport system and passive diffusion [8]. Magnesium absorption depends on the intake. Pharmacological doses of vitamin D increase magnesium absorption in vitamin D deficiency. However, magnesium absorption is independent of vitamin D levels. Additionally, vitamin D may reduce magnesium retention through increases in urinary magnesium excretion. Intestinal interactions between magnesium and calcium or phosphate have been demonstrated [8]. In the gut, calcium affects magnesium absorption and vice versa; a high calcium intake can reduce magnesium absorption and a low magnesium intake can increase calcium absorption. Parathyroid hormone responds to an increase in magnesium absorption. The exact mechanisms of these interactions are unclear. In 2017, the American College of Rheumatology recommended a daily dose of 600-800 IU/day of vitamin D and 1,000-2,000 mg/day of calcium along with lifestyle modifications [9]. If long-term steroid use is necessary, vitamin D and magnesium supplementation should be considered.

## Conclusions

The key message for clinicians is that vitamin D supplements should be administered daily in patients who are receiving glucocorticoid therapy. Furthermore, it is important to correct electrolyte imbalance in patients who suffer from ventricular arrhythmia with prolonged QT because it is helpful in early resolution and prevention of recurrence.
